# Relief Role of Lysine Chelated Zinc (Zn) on 6-Week-Old Maize Plants under Tannery Wastewater Irrigation Stress

**DOI:** 10.3390/ijerph17145161

**Published:** 2020-07-17

**Authors:** Rehan Ahmad, Wajid Ishaque, Mumtaz Khan, Umair Ashraf, Muhammad Atif Riaz, Said Ghulam, Awais Ahmad, Muhammad Rizwan, Shafaqat Ali, Saad Alkahtani, Mohamed M. Abdel-Daim

**Affiliations:** 1 Department of Environmental Sciences, Gomal University, Dera Ismail Khan 29050, Pakistan; malikrehan2@yahoo.com (R.A.); mumtazmarwat@yahoo.com (M.K.); 2 Nuclear Institute for Agriculture and Biology (NIAB), Faisalabad 38000, Pakistan; raoumar05@yahoo.com (W.I.); atif_riaz@hotmail.com (M.A.R.); 3 Department of Botany, Division of Science and Technology, University of Education, Lahore 54770, Pakistan; umairashraf2056@gmail.com; 4 Department of Soil Science, Gomal University, Dera Ismail Khan 29050, Pakistan; saidghulam63@yahoo.com; 5 Department of Applied Chemistry, Government College University, Faisalabad 38000, Pakistan; awaisahmed@gcuf.edu.pk; 6 Department of Environmental Sciences and Engineering, Government College University Allama Iqbal Road, Faisalabad 38000, Pakistan; mrazi1532@yahoo.com; 7 Department of Biological Sciences and Technology, China Medical University, Taichung 40402, Taiwan; 8 Department of Zoology, College of Science, King Saud University, P.O. Box 2455, Riyadh 11451, Saudi Arabia; salkahtani@ksu.edu.sa (S.A.); abdeldaim.m@vet.suez.edu.eg (M.M.A.-D.); 9 Pharmacology Department, Faculty of Veterinary Medicine, Suez Canal University, Ismailia 41522, Egypt

**Keywords:** biofortification, maize, zinc, chromium, zinc-lysine, oxidative stress, tannery wastewater

## Abstract

Tannery wastewater mainly comes from leather industries. It has high organic load, high salinity, and many other pollutants, including chromium (Cr). Tannery wastewater is generally used for crop irrigation in some areas of Pakistan and worldwide, due to the low availability of good quality of irrigation water. As tannery wastewater has many nutrients in it, its lower concentration benefits the plant growth, but at a higher concentration, it damages the plants. Chromium in tannery wastewater accumulates in plants, and causes stress at physiological and biochemical levels. In recent times, the role of micronutrient-amino acid chelated compounds has been found to be helpful in reducing abiotic stress in plants. In our present study, we used lysine chelated zinc (Zn-lys) as foliar application on maize (*Zea mays* L.), growing in different concentrations of tannery wastewater. Zinc (Zn) is required by plants for growth, and lysine is an essential amino acid. Maize plants were grown in tannery wastewater in four concentrations (0, 25%, 50%, and 100%) and Zn-lys was applied as a foliar spray in three concentrations (0 mM, 12.5 mM, and 25 mM) during plant growth. Plants were cautiously harvested right after 6 weeks of treatment. Foliar spray of Zn-lys on maize increased the biomass and improved the plant growth. Photosynthetic pigments such as total chlorophyll, chlorophyll a, chlorophyll b and contents of carotenoids also increased with Zn-lys application. In contrast to control plants, the hydrogen peroxide (H_2_O_2_) contents were increased up to 12%, 50%, and 68% in leaves, as well as 16%, 51% and 89% in roots at 25%, 50%, and 100% tannery water application, respectively, without Zn-lys treatments. Zn-lys significantly reduced the damages caused by oxidative stress in maize plant by decreasing the overproduction of H_2_O_2_ and malondialdehyde (MDA) in maize that were produced, due to the application of high amount of tannery wastewater alone. The total free amino acids and soluble protein decreased by 10%, 31% and 64% and 18%, 61% and 122% at 25%, 50% and 100% tannery water treatment. Zn-lys application increased the amino acids production and antioxidant activities in maize plants. Zn contents increased, and Cr contents decreased, in different parts of plants with Zn-lys application. Overall, a high concentration of tannery wastewater adversely affected the plant growth, but the supplementation of Zn-lys assertively affected the plant growth and enhanced the nutritional quality, by enhancing Zn and decreasing Cr levels in plants simultaneously irrigated with tannery wastewater.

## 1. Introduction

Maize (*Zea mays* L.) is one of the main crop of Pakistan and worldwide. It is usually sown in April-June, and harvesting is done in October [1]. In Pakistan, maize is vital cereal crop after rice and wheat. [2]. It is of high nutritional value, containing protein, starch, fiber and other micronutrients [3]. There are almost 800 tanneries in Pakistan [4], and these tanneries contain high levels of chromium (Cr), and other nutrients, such as potassium dichromate, are used during the tanning process [5]. Tannery industries release waste containing toxic chemicals, including high amounts of sulfides, Cr and many other chemicals [6,7]. Tannery wastewater is used for irrigation in Pakistan, as it has nutrients that promote plant growth. At low concentration, tannery wastewater promotes plant growth, whereas at high concentration, it induces toxicity in plants. As tannery wastewater contains a high amount of iron, manganese, zinc (Zn), Cr, alkanity, COD (chemical oxygen demand), BOD (biological oxygen demand) and total dissolved salts, irrigation causes the severe damage in plants. At high concentrations, tannery wastewater can decrease the plant biomass and seed production. length of root and shoot, root and shoot dry biomass, total leaf area, chlorophyll a, chlorophyll b, total chlorophyll, protein and amino acids are also affected with tannery wastewater [8,9]. Many studies have reported that Cr caused negative effects on plants, and results in oxidative stress and growth inhibition [10,11]. Cr induced the lipid peroxidation in plants which results in harm to cell membranes. This also results in the degradation of photosynthetic pigments in plants, which also affect plant growth [12,13,14]. Irrigation with tannery wastewater may decrease photosynthetic pigments in plants prominently chlorophyll synthesis. Chromium toxicity in plants reduced the photosynthesis activity of plants, and it also changed the chloroplast structure [15,16,17].

Foliar application is a method of supplying nutrients to plants by direct spraying them in liquid form on leaves. Nutrients applied through the foliage spray are absorbed more rapidly than soil application [18]. Zinc is an essential metal for plant metabolism, if present in trace amounts [19]. At low levels, Zn supply to plants increase the activity of enzymes, such as superoxide dismutase (SOD), peroxidase (POD), catalase (CAT), and ascorbate peroxidase (APX) in the plants [20]. Zinc is among those elements whose presence is crucial for proper growth of plants and humans beings. Zinc containing enzymes belongs to all six classes of enzymes: transferases, hydrolases, ligases, oxidoreductases, lyases, and isomerases [21,22]. In regulatory proteins, Zn plays a structurally important part [23]. It also helps in regulating the protein and carbohydrate metabolism in plants [24]. It has been observed that the foliar application of Zn on plants improved the growth and development of plants [25]. It has been reported that the application of foliar Zn resulted in high hormone contents in bean plants. This can be due to the fact that Zn helps in building up the natural auxin, such as indole-3-acetic acid (IAA), which leads to the galvanization of the cell division and enlargement [26]. The application of Zn, along with other nutrients, is found to be useful for plants, as reported previously [27]. Zn chelated amino acids are better for crop growth, and increase the yield of plants [28]. Lysine is an essential amino acid, but living organisms cannot synthesize it in their body; it has to be taken from an outside source [29]. Lysine makes proteins, and it also enhances the nutritional quality of plants [30]. It has been observed that zinc-lysine (Zn-lys) foliar spray has prominently amplified Zn contents, photosynthetic rate, grain yield and enzyme activities in different plant tissues [31]. Plant height, gas exchange attributes and photosynthetic activities were increased in rice by the application of Zn-lys [32]. Maize is an important crop for humans and animals. Previously, no study has reported the effects of Zn-lys on maize crop irrigated with tannery wastewater. Thus, in this experiment, we used lysine chelated Zn as foliar spray on maize irrigated with tannery wastewater, to see the possible positive effects of this chelated compound on the properties of plant growth and photosynthetic content. The central aims of the study were (1) to assess the toxic effects of tannery wastewater in maize and monitor uptake of Cr in different parts of maize, (2) to determine the effects of Zn-lys foliar spray on maize growth that was irrigated with tannery wastewater, and finally (3) to increase growth, photosynthetic content and antioxidative properties of maize with the application of Zn-lys irrigated with tannery wastewater.

## 2. Materials and Methods

### 2.1. Experimental Site

Experiment was carried out in botanical garden of Government College University, Faisalabad (GCUF). While the analysis were carried out in the Nuclear Institute for Agriculture and Biology (NIAB) Faisalabad, Pakistan.

### 2.2. Pot Experiment

Preparation of soil for growing maize was the first step of the experiment. Soil was air dried for one week without direct sunlight and then it was sieved through a 30 mm sieve. Sieved soil was then filled in pots, and the average weight of soil in each pot was 8 kg. The physio-chemical properties of soil have been shown in Table 1. Maize seeds for sowing were obtained from Ayub Agricultural Research Institute, Faisalabad. These seeds were washed with distilled water before sowing in plastic pots.

### 2.3. Treatments

Plants were irrigated with tannery wastewater, in concentrations of 0% 25% 50%, and 100% and sprayed with three different concentrations of Zn-lys (0 mM, 12.5 mM, 25 mM). The properties of tannery wastewater have been shown in Table 2. Three replicates for each treatment were done. Pots arrangement was according to complete randomized design with factorial arrangement. Zn-lys was synthesized by following the standard procedure [33] by using zinc sulfate heptahydrate and L-lysine monohydrochloride, as Zn and lysine salts, respectively.

### 2.4. Analysis

#### 2.4.1. Leaf Area

Leaf area was measured by leaf meter after six weeks of treatment.

#### 2.4.2. Chlorophyll Contents Determination

Leaf samples were collected after 6 weeks of treatment. Chlorophyll a, chlorophyll b, total chlorophyll and carotenoid contents were determined by using a spectrophotometer (Halo DB-20/DB-20S, Dynamica Company, London, UK) [34]. The extended top leaves were firstly weighed on an electric balance and then dipped in 85% (v/v) aqueous acetone, under dark conditions. The supernatant was collected and centrifuged at 4000× *g* rpm for ten minutes. In order to make spectrophotometric measurements feasible, 85% aqueous acetone was added for dilution. The measurements were taken at three different absorbances, of 452.5, 644 and 663 nm, against a blank (85% liquid acetone). The following formulae were used for the calculation of photosynthetic pigments:

For calculation of Chlorophyll a: Chlorophyll a (µg·mL^−1^) = 10.3 × E_663_ − 0.98 × E_644_

For calculation of Chlorophyll b: Chlorophyll b (µg·mL^−1^) = 19.7 × E_644_ − 3.87 × E_663_

For calculation of Total chlorophyll: Total chlorophyll = chlorophyll a + chlorophyll b

For calculation of Total carotenoids (µg·mL^−1^) = 4.2 × E_452.5_ − {(0.0264 × chl a) + (0.426 × chl b)}

All of these pigment fractions were calculated as fresh weight in mg g^−1^.

#### 2.4.3. Free Amino Acids Determination

Free amino acid was calculated by following the procedure of Hamilton et al. [35] Firstly, 0.1 g of fresh leaf was grinded in buffer solution, with a pH of 7.0. This mixture was then centrifuged for ten minutes at 8000× *g* rpm. Then, 1.0 mL of supernatant + 1.0 mL of 10% pyridine + 1.0 mL of 2% ninhydrine were mixed in test tubes. This solution was then heated for thirty minutes in a bath of boiling water, and finally, the violet color was obtained. Then, the solution was diluted with distilled water, and the absorbance at 570 nm was measured on a spectrophotometer.

#### 2.4.4. Analysis of Antioxidant Enzymes

Spectrophotometer was used to determine the antioxidant enzyme’s activities, including SOD, peroxidase (POD), CAT, and APX, in roots and leaves of maize plant.

Fresh roots and leaf samples (0.5 g each) were taken after six weeks of treatments. These samples were ground in beads beater, and then the samples were homogenized in phosphate buffer (0.05 M), having pH 7.8 in chilled conditions. Supernatant was centrifuged at 12,000× *g* for duration of 10 min at 4 °C. SOD, POD activities and malondialdehyde (MDA) contents were measured using the methods of Zhang [36].

The method of Aebi [37] was taken up for catalase (CAT, EC 1.11.1.6) activity evaluation. The 3.0 mL reaction mixture was comprised of H_2_O_2_ (300 mM) 100 μL and enzyme extract 100 μL, which was ground in buffer containing a volume of 2.8 mL phosphate buffer (50 mM) with 2.0 mM EDTA (pH 7.0). Catalase activity was calculated by assessing the absorbance reduciton on a spectrophotometer at 240 nm, as a result of the disappearance of hydrogen peroxide (ε = 39.4 mM^−1^·cm^−1^).

The method of Nakano and Asada [38] was used for finding out of ascorbate peroxidase (APX, EC 1.11.1.11) activity. The reaction solution consisted of 100 μL ascorbate (7.5 mM), 100 μL H_2_O_2_ (300 mM), 100 μL enzyme extract, and 2.7 mL potassium phosphate buffer (25 mM), with 2.0 mM EDTA having a pH of 7.0. The oxidation of ascorbate was measured by the change in absorbance at 290 nm in a spectrophotometer (ε = 2.8 mM^−1^·cm^−1^).

#### 2.4.5. Determination of Soluble Protein

Method of Bradford [39] was used to determine the soluble protein contents in leaves. The dye used was albumin and Coomassie Brilliant Blue G-250, was the standard. In liquid nitrogen, frozen samples of leaves (0.5 g) were crushed with a pestle and mortar. Afterwards, these were homogenized in 50 mm sodium phosphate buffer (10 mL) having 7.0 pH, and which also contains l.0 mm EDTA-Na_2_ and polyvinyl pyrrolidine-40, also known as PVP-40, 2% (w/v). Additionally, the homogenate was centrifuged for duration of 15 min at 11,000× *g* at 4 °C. After that, the supernatant was used for analyzing the activity of an antioxidant enzyme. Furthermore, the addition of of Bradford solution (1 mL) was added in 100 µL crude extract. The absorbance was then measured at 595 nm wavelength for the determination of the entire protein content. A standard curve of BSA was used for determining the protein concentration.

#### 2.4.6. Contents of Hydrogen Peroxide

To observe the H_2_O_2_ content in plants, 50 mg of fresh leaf and root was used and grinded in 3 mL of phosphate buffer (50 mM), having a pH of 6.5, and further centrifuged at 6000× *g* for a duration of 25 min.

In order to find out the H_2_O_2_ content, the extracted solution in a quantity of 3 mL was mixed, with 1 mL of 0.1% titanium sulphate in 20% (v/v) H_2_SO_4_, and then it was centrifuged at 6000× *g* for a 15-min duration. At 410 nm, the intensity of yellow color of the supernatant was determined. H_2_O_2_ content was measured with the use of the extinction coefficient (0.28 µmol^−1^·cm^−1^).

#### 2.4.7. Chromium and Zn Concentration Evaluation

With all the three replications, the harvesting of maize plants was done after six weeks. They were then washed extensively with water, distilled water, and finally with the deionized water, to clean them. Leaves, roots and stems were separated from the main plant body. Plant biomass was first dried for 48 h in an oven, with a temperature of 80 °C. The dried biomass was then ground into powder. Each sample was taken in 0.5 g quantity in 100 mL flasks, and 15 mL of concentrated HNO_3_ were added in each flash using a pipette. The acid and samples were then mixed, and then the flasks were placed on a hot plate. The temperature was slowly increased up to 275 °C, which resulted in the formation of thick yellow fumes in the flask. when these fumes became low in quantity, then hydrogen peroxide was added carefully in flasks, until dense yellow fumes totally vanished. The flasks were then removed from the hot plate, and these were shifted to the lab, where they became colorless. Their volume was made up to 25 mL with distilled water. For the measurement of the concentration of the Cr and Zn contents in different plant parts, flame atomic absorption spectrometry (Nova 400 Analytik Jena, Jena, Germany) was used. For quality control and quality assurance (QA/QC), for Cr and Zn determination in the plant samples, blanks were placed parallel to the samples, containing acids without samples, and were digested in a similar manner to sample digestions, and these were also analyzed for Cr and Zn, and the calculations were adjusted accordingly. The standards of Cr and Zn with different known concentrations were also run on the instrument, and random standards were also analyzed after every ten samples during analyses, to ensure authenticity of the analyses.

#### 2.4.8. Statistical Analysis

The analysis of variance (ANOVA) was carried out by using a statistical package. SPSS version 16.0 (SPSS, Chicago, IL, USA). For the determination of the significant difference between values, Tukey’s test was used.

## 3. Results

### 3.1. Effects on Plant Growth and Yield

**Figure 1:**
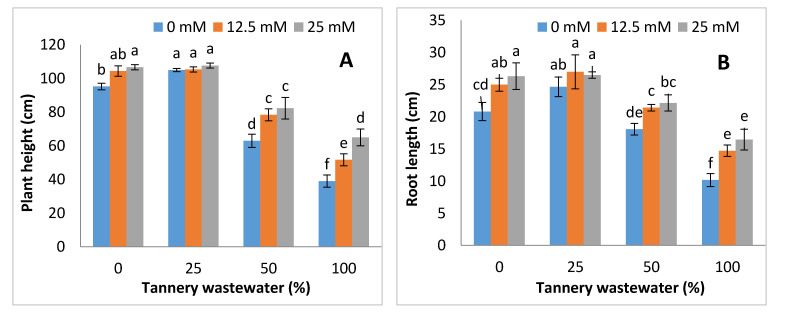

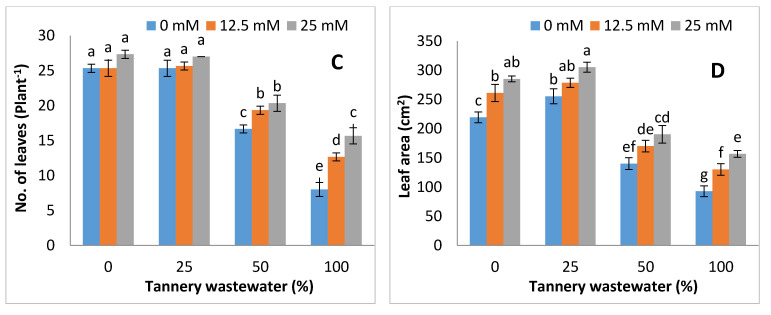
Graphs showing the effect of foliar spray of zinc chelated lysine (0, 12.5, 25 mM) on plant height (**A**), root length (**B**), number of leaves (**C**) and leaf area (**D**) of maize plants growing under 0, 25, 50, and 100% concentration of tannery wastewater. Values are means of three replicates. Statistical analyses were carried out by ANOVA, followed by Tukey’s Post-Hoc test.

The application of tannery wastewater to maize substantially reduced the morphological growth of maize (Figure 1). However, Zn-lys application modulated the growth under contaminated soil conditions. For instance, on average, the plant height, root length, number of leaves, and leaf area decreased up to 27% and 49%, 15% and 43%, 28% and 53%, and 35% and 50%, at 50% and 100% tannery wastewater, compared with the control. The average values for all morphological parameters were recorded as higher and/or similar, but not up to a significant level at 25% tannery wastewater, as compared with control. Moreover, with increasing tannery water treatment percentage in the irrigation water, the effects of Zn-lys were more prominent as compared to lower doses of tannery wastewater, as such effects were also more positive, with an increased Zn-lys application dose. Moreover, 100% tannery wastewater treatment with no Zn-lys spray showed the lowest growth and biomass, including root length, plant height, leaf area and number of leaves per plant Figure 1).

**Figure 2:**
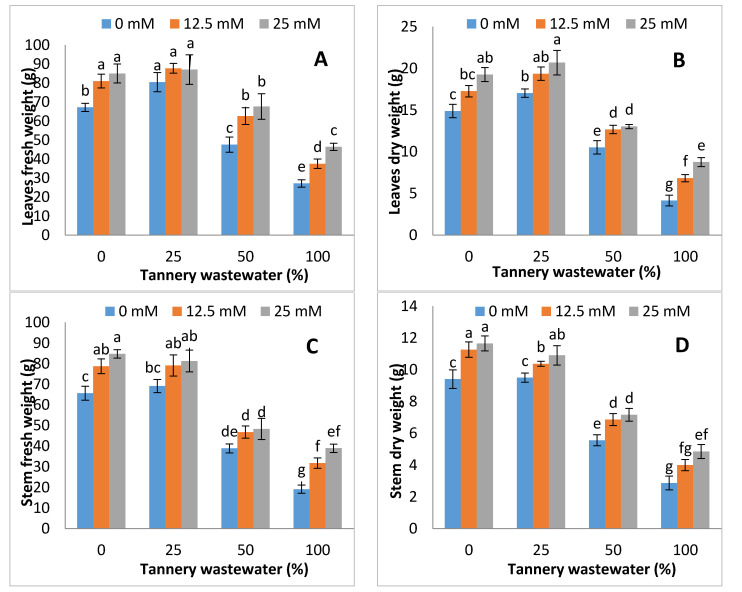

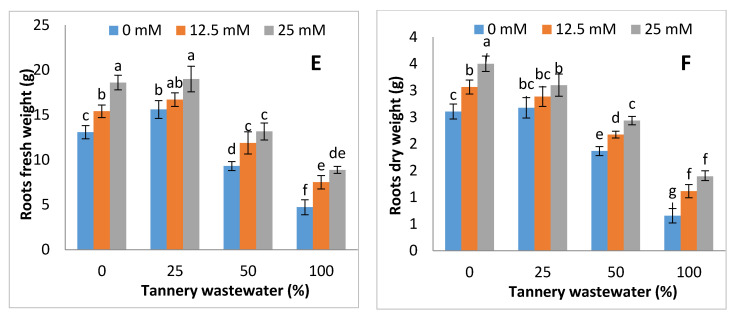
Graphs showing effect of foliar spray of zinc chelated lysine (0, 12.5, 25 mM) on fresh and dry biomass of leaves (**A**,**B**), stem (**C**,**D**) and roots (**E**,**F**) of maize plants growing under 0%, 25%, 50%, 100% concentration of tannery wastewater. Values are means of three replicates. Statistical analyses were carried out by ANOVA, followed by Tukey’s Post-Hoc test.

The biomasses of stem, leaves and roots (fresh and dry) were substantially lowered under tannery wastewater application, and such reductions were more severe, with increasing the tannery wastewater concentration, except at 25%, whilst exogenous Zn-lys application improved the fresh and dry biomass, either with or without tannery wastewater treatments. The highest fresh and dry biomass was recorded in Zn-lys application at 25 mM dose under control, and each tannery wastewater treatment (Figure 2).

### 3.2. Effects on Photosynthetic Pigments

**Figure 3:**
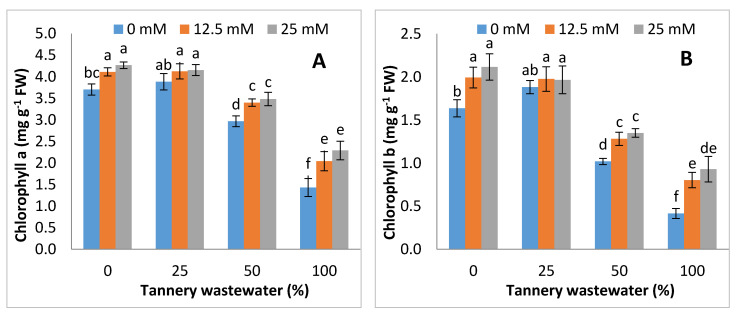

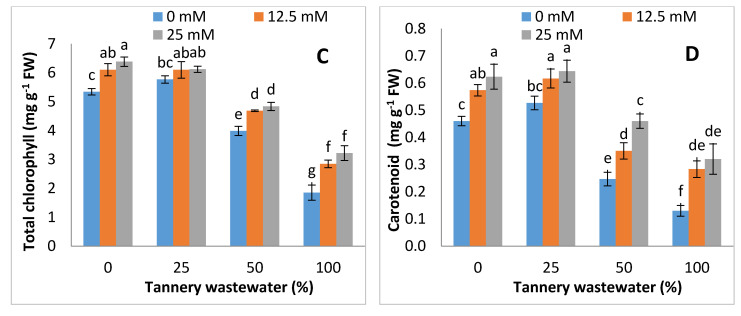
Graphs showing effect of foliar spray of zinc chelated lysine (0, 12.5, 25 mM) on Chlorophyll a (**A**), Chlorophyll b (**B**), total Chlorophyll content (**C**) and carotenoids (**D**) of Maize plants, growing under 0, 25, 50, 100% concentration of tannery wastewater. Values are means of three replicates. Statistical analyses were carried out by ANOVA, followed by Tukey’s Post-Hoc test.

The photosynthetic pigments, i.e., chlorophyll a, chlorophyll b, total chlorophyll contents and carotenoids, decreased with increasing concentrations of tannery wastewater, especially as the extent of such reductions were obviously lower in Zn-lys applied plants than non-applied plants. The application of tannery wastewater specifically at 50% and 100% led to 18% and 52%, 36% and 63%, 24% and 56% and 36% and 56% reductions in Chl a, Chl b, total Chl contents and carotenoids, respectively. The values were marginally different than for the control at 25% wastewater treatment. The lowest values for Chl a, Chl b, total Chl contents and carotenoids under each wastewater treatment were recorded in plants without Zn-lys application, whilst Zn-lys application at 25 mM led to a prominent increase in photosynthetic pigments, as compared with non-Zn-lys applied plants (Figure 3).

### 3.3. Effects on H_2_O_2_ and MDA Production

**Figure 4:**
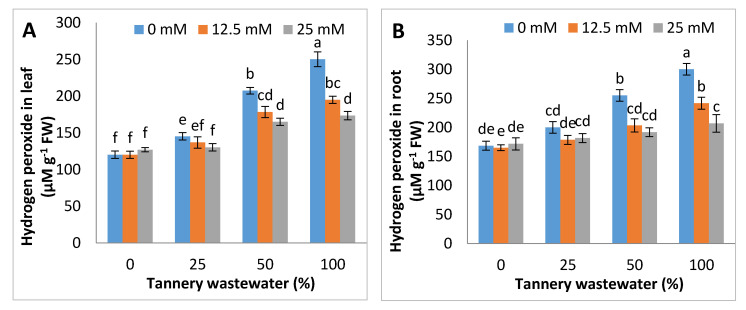

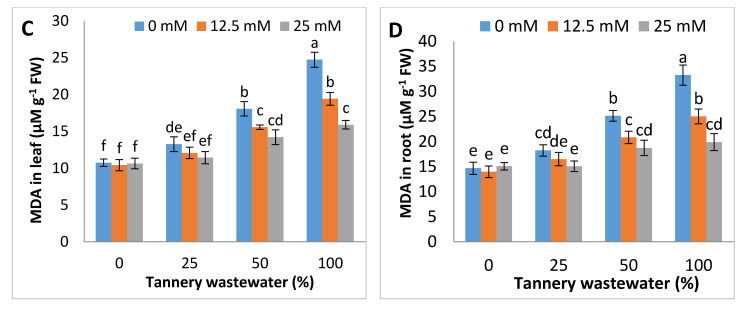
Graphs showing effect of foliar spray of zinc chelated lysine (0, 12.5, 25 mM) on hydrogen peroxide and MDA in leaves (**A**,**C**) and roots (**B**,**D**) of maize plants, growing under 0%, 25%, 50%, 100% concentration of tannery wastewater. Values are means of three replicates. Statistical analyses were carried out by ANOVA, followed by Tukey’s Post-Hoc test.

Tannery water application caused a remarkable increase in the H_2_O_2_ and MDA contents in the leaves and root of maize. As compared to the control, content of H_2_O_2_ and MDA were increased by up to 12%, 50%, and 68%, and 11%, 29% and 48%, as well as 16%, 51% and 89% and 14%, 48% and 79% in leaves and root, at 25%, 50%, and 100% tannery water application, respectively. Moreover, Zn-lys treatments led to a substantial reduction in H_2_O_2_ and MDA in leaves and root of maize than non- Zn-lys applied plants, whilst the H_2_O_2_ and MDA contents gradually lowered, with an increase in the concentration of Zn-lys (Figure 4).

### 3.4. Effects on Free Amino Acids, Soluble Protein, Soluble Sugar and Free Proline

**Figure 5:**
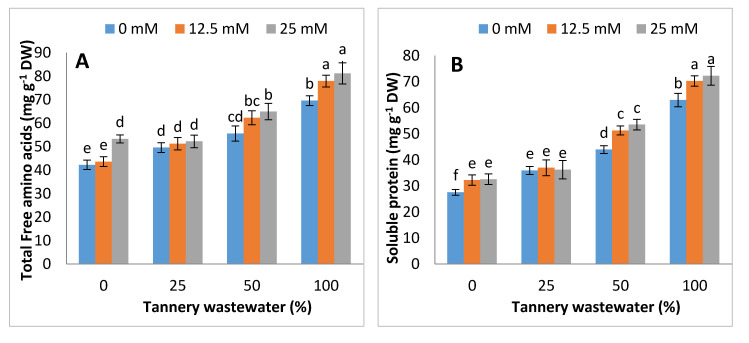

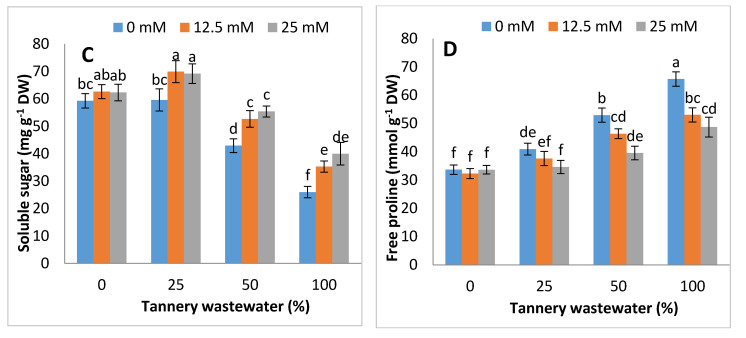
Graphs showing effect of foliar spray of Zn chelated lysine (0, 12.5, 25 mM) on total free amino acids (**A**), soluble protein (**B**), soluble sugar (**C**) and free proline (**D**) in maize plants, growing under 0%, 25%, 50%, 100% concentration of tannery wastewater. Values are means of three replicates. Statistical analyses were carried out by ANOVA, followed by Tukey’s Post-Hoc test.

The up-regulations in concentration of the soluble protein and total free amino acids were noticed with the foliar application of Zn-lys, under tannery wastewater treatment in maize plants. Compared with control (0% wastewater treatment), the total free amino acids and soluble protein contents gradualy increased by 10%, 31% and 64%, and 18%, 61% and 122%, at 25%, 50% and 100% tannery water treatment. In addition, the soluble sugars increased by 8% at 25% tannery wastewater, whilst being reduced by 18% and 45% at 50% and 100% tannery wastewater, respectively. On the other hand, the free proline contents increased with the progression of the application dose of tannery wastewater, i.e., increased by 14%, 39%, and 68% at 25%, 50% and 100% tannery wastewater, as compared with control. Overall, total free amino acids, soluble sugars, soluble protein and free proline were examined to be high in plants treated with the foliar spray of Zn-lys, as compared to plants without any Zn-lys application (Figure 5).

### 3.5. Effects on Antioxidant Enzymes Activities

**Figure 6:**
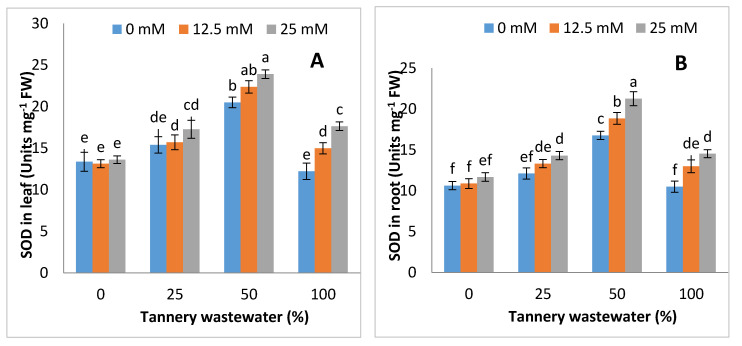

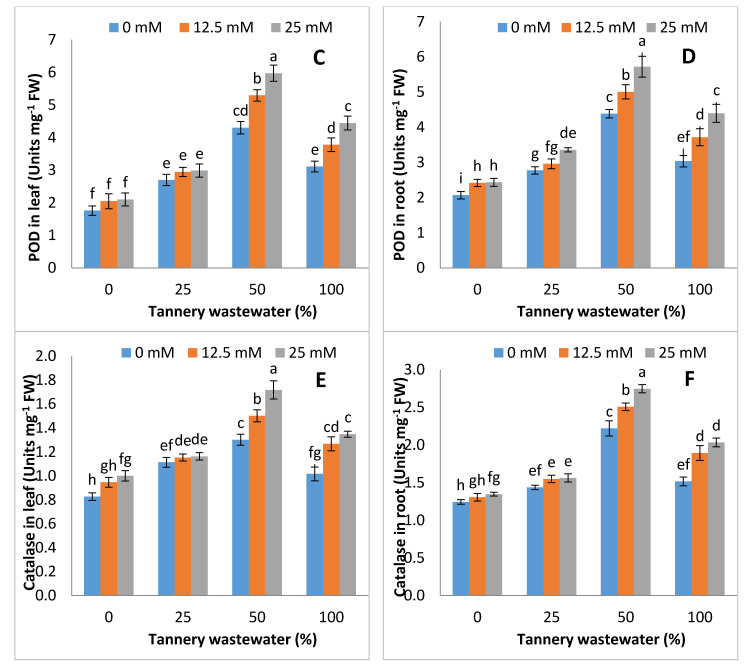
Graphs showing effect of foliar spray of zinc chelated lysine (0, 12.5, 25 mM) on superoxide dismutase (SOD), peroxidase (POD) and catalase (CAT), in leaves (**A**,**C**,**E**) and roots (**B**,**D**,**F**) of maize plants growing under 0%, 25%, 50%, 100% concentration of tannery wastewater. Value are means of three replicates. Statistical analyses were carried out by ANOVA, followed by Tukey’s Post-Hoc test.

The irrigation with tannery water altered the antioxidant enzyme activities, including SOD, POD and CAT, in both the leaves and root of maize. Overall, the antioxidant activities were increased from 0 to 50% application of wastewater, followed by an abrupt decline at 100% wastewater treatment, in both leaves and root of maize plants. On the other hand, the application of Zn-lys at all wastewater treatments improved the antioxidant enzyme activities, whereas the antioxidant activities were remained the maximum at the highest level of Zn-lys application (Figure 6).

Furthermore, the lowest antioxidant enzyme activities were found in the control (without wastewater treatment and Zn-lys application) for both leaves and root of maize plants (Figure 6).

### 3.6. Effects on Zn and Cr Uptake

**Figure 7:**
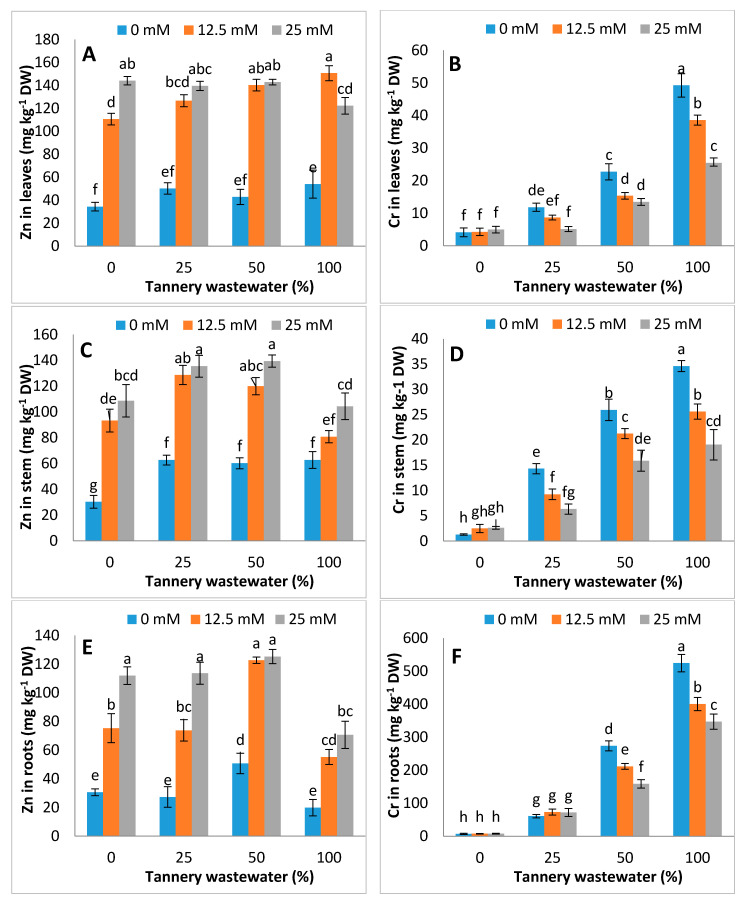
Graphs showing effect of foliar spray of zinc chelated lysine (0, 12.5, 25 mM) on Cr and Zn concentration in leaves (**A**,**B**), stem (**C**,**D**) and roots (**E**,**F**) of maize plants growing under 0%, 25%, 50%, 100% concentration of tannery wastewater. Values are means of three replicates. Statistical analyses were carried out by ANOVA, followed by Tukey’s Post-Hoc test.

The application of Zn-lys increased the Zn contents in leaves, stem and roots of maize, whilst it reduced the Cr contents in respective plant parts substantially under tannery wastewater application, as the tannery wastewater had been added, which led to an increase in the concentration of Cr. As the tannery wastewater volume increased, the concentration of Cr also increased, like a dose-additive phenomena which led to contamination of the plant tissues with Cr. Zn-lys (12.5, 25 mM) helped the plants to decrease the contamination of Cr. Thus, the Cr concentrations decreased by using Zn-lys at each tannery wastewater treatment. Due to the addition of Zn-lys, Zn concentration was increased in all parts of maize plant (Figure 7).

## 4. Discussion

Tannery wastewater often consists of various toxic metals that could severely affect the morpho-physiological growth of crop plants. The application of tannery wastewater to crops could contaminate agricultural lands, as well as food products. When edible plants are grown in contaminated soil, many heavy metals can accumulate in plant parts, which are toxic to plants and also cause harm to the health of human beings [10,40,41]. The tannery wastewater used in the present study has higher values of Cr, and other different parameters (Table 2). Previous study also reported the higher values of Cr, and other studied attributes of tannery wastewater used for the irrigation of plants [42]. The higher values of COD and TOC (Total organic carbon) of tannery wastewater may also cause negative effect on plant growth, which might be due to the alteration of nutrient dynamics in soil and their uptake by plants [15,42,43]. It has been reported that the removal of COD and TOC from tannery wastewater is a difficult task and requires suitable techniques [44]. Thus, most of the industries are discharging wastewater without any proper treatment, and farmers are using this unsuitable water without any treatment. Present study investigated the effects of tannery wastewater on morpho-physiological features of maize plants, and the part of Zn-lys application in modulating the early growth and related physio-biochemical attributes of maize plants. Tannery wastewater application reduced the growth attributes, as well as the biomass of maize (both fresh and dry) plants considerably, whereas Zn-lys application improved the early growth and biomass accumulation of maize (Figure 1 and Figure 2). Tannery wastewater related decline in growth may possibly be due to the pools of heavy metals, especially Cr in the tannery wastewater, that may not only interfere with the plant nutrient uptake, but also disrupt the normal plant metabolism. The Zn-lys application might increase the uptake of more nutrients from the soil, and provide relief against Cr stress. Moreover, comparatively higher growth and biomass accumulation was observed at a lower concentration of tannery wastewater, i.e., 25%, than other concentrations applied in the study. Lower concentrations of tannery wastewater could promote the plant growth, as it contains differential amounts of micro- and macro-nutrients, and could also be a good fertilizer source. Previously, Calheiros et al. [15] reported that the application of tannery wastewater at lower doses showed the positive effects on growth related attributes of *Trifolium pretense*. On the other hand, the growth and biomass accumulation of *Spirodela polyrrhiza* was substantially reduced under higher doses of tannery wastewater application [45]. Moreover, Zn-lys being an amino-chelated fertilizer could hinder the mobility of otherwise readily available Cr, as it forms complexes with various heavy metals, including Cr [46]. Zn-lys application induced the improvement in growth and biomass accumulation under higher doses of tannery wastewater, which was also found in wheat [47] and rice [43] previously.

The chlorophyll contents in plants applied with tannery water were considerably lower, whilst the foliar application of Zn-lys modulated the leaf chlorophyll contents of maize (Figure 3). Metallic ions (ie. Cr) present in leather tannery wastewater showed enormous interference with different vital micronutrients that eventually resulted in the subsequent degradation of chlorophyll contents [48,49]. Cr-induced reductions in chlorophyll biosynthesis were previously reported in different crops, like maize, barley, and spinach [42,50,51]. An enhancement in the chlorophyll contents in Zn-lys applied plants may be ascribed to the higher Zn contents, with subsequent decreases in Cr uptake and accumulation in Zn-lys applied plants, as Zn is actively involved in the structural and functional integrity of chloroplast ultra-structures and chlorophyll biosynthetic in plants [52,53]. Zn-induced improvements in the chlorophyll contents were also observed in *Raphanus sativus* [54] and wheat [47].

The application of tannery wastewater resulted in significant increase in the production of H_2_O_2_ and MDA levels, whereas exogenous Zn-lys application relieved the oxidative damange by reducing the over-production of H_2_O_2_ and MDA contents in growing maize plants (Figure 4). The uptake of various metallic ions present in tannery waste most often leads to the over-production of ROS, and hence is the cause of oxidative stress in plants. Increased MDA contents exhibited the higher lipid per-oxidation rate, whereas Cr, being a potent heavy metal in tannery waste, also generates the free radicals that oxidize biological membranes by reacting with them, and hence caused structural and functional damage to plants [10,42]. On the other hand, Zn-lys proved to be a stress alleviator, as it not only reduced the Cr uptake, but also had a ROS scavenging role. Previously, Mohammadi et al. [23] reported that the application of amino-acids chelated Zn helped in the reduction of oxidative stress in lettuce.

Regulation in osmolyte accumulation and anti-oxidative enzyme activities are an innate resonse of the plan towards stress conditions. This study reported the rise in total free amino acids, soluble proteins and free prolines, with a decrease in soluble sugars in tannery wastewater treated plants, whereas the contents of soluble protein, total free amino acids and soluble sugars were comparatively higher in Zn-lys applied plants than non-Zn-lys applied plants (Figure 5). Furthermore, the antioxidant activities, i.e., SOD, POD and CAT, were increased from 0 to 50% application of tannery wastewater, followed by a sudden decline at full dose (100%) of wastewater treatment. Unexpected severe oxidative stress resulted in decline of activities related to the antioxidant enzymes as studied by Adrees et al. [55,56]. Comparable results were observed by Dey et al. [57] Overall, Zn-lys application improved the antioxidant activities in plants, both under tannery wastewater and control treatments (Figure 6). Increased soluble sugars and proline might be an innate response of plants against tannery water-induced stress conditions. On the other hand, heavy metals may disrupt amino acids and proteins, thus causing structural and functional damage to the plants. Plant often accumulates differential amounts of various osmolytes and proteins, in stress conditions that not only protect the biological structures, but also help in the cleansing of ROS [41]. Moreover, Zn-lys application improved the antioxidant enzyme activities, and thus, decreased the subsequent oxidative stress in various plant system, as specified by the decline of H_2_O_2_ and MDA contents. Recently, Zaheer et al. [42] reported that the application of Zn-lys chelated fertilizer improved the POD, SOD and CAT activities in roots and leaf of spinach grown in tannery wastewater contaminated soil. The SOD detoxifies O_2_^−^ to O_2_ and H_2_O_2_, whereas CAT and POD converted it to H_2_O and O_2_ [58]. This antioxidant-related ROS quenching mechanism, that was further enhanced by Zn-lys application, might help the plants to better tolerate the tannery wastewater related stress conditions. Exogenous Zn application at 25 μM enhanced the antioxidant activities significantly in bean plants [59]. In addition, Zn-lys application enhanced the Zn contents in different plant parts of maize, whereas it reduced the Cr concentrations in the subsequent plant parts (Figure 7). Higher concentrations of Cr in plants without Zn-lys may be related to the higher doses of tannery wastewater application, whereas amino-acid chelated Zn has the capability to form complexes with various metallic ions, and thus reduced their uptake and translocation in different plant organs [60]. Furthermore, the reductions in the Cr contents in lysine-chelated Zn applied plants were previously noted in rice, wheat and spinach [42,43]. Overall, the Zn-lys application improved the early growth, biomass accumulation, physio-biochemical attributes and Zn contents in different plant parts, whilst it reduced the Cr contents.

## 5. Conclusions

In conclusion, tannery wastewater proved beneficial to maize plants if applied at a low concentration, but at higher doses, it reduced the morphological growth and photosynthetic pigments of plants, whilst it enhanced the oxidative stress in terms of H_2_O_2_ and MDA contents. Moreover, important activities of vital antioxidant enzyme including SOD, CAT and POD, in the roots and leaves of maize plant were enhanced with the application of tannery water as compared with the control, which has been further modulated by Zn-lys application. In addition, foliar Zn-lys application reduced the Cr contents in different parts of maize plants, while Zn contents were improved under these treatments. Hence, tannery wastewater can be applied as a fertilizer at lower doses (as it contains some micro/macro elements), however, the higher levels may be toxic to maize plants for normal growth. The dose of Zn-lys should be optimized if it needs to be used as a fertilizer source. Lysine chelated Zn can be efficiently used to improve the growth and stress reduction in maize plants, if applied in a suitable quantity to plants growing in tannery wastewater.

## Figures and Tables

**Table 1:** **Physiochemical** Properties of soil for experiment.

Physicochemical Properties	
Texture	Clay loam
pH (1/2.5 soil to water ratio)	6.7
Sand (%)	27
Silt (%)	20
Clay (%)	53
EC_e_ (Electric conductivity) (dS·m^−1^)	2.9
SAR (Sodium adsorption ratio) (mmol^−1^)^1/2^	6.5
Organic matter (%)	0.31
Cl^−^ (mmol·L^−1^)	2.34
Available Cu^2+^ (mg·kg^−1^)	0.35
Available Zn^2+^ (mg·kg^−1^)	0.85
Na^2+^ (mmol·L^−1^)	3.7
HCO_3_ (mmol·L^−1^)	3.55
Available P (mg·kg^−1^)	2.17
K^+^ (mmol·L^−1^)	0.06
SO_4_^2−^ (mmol·L^−1^)	6.67
Ca^2+^ + Mg^2+^ (mmol·L^−1^)	3.5

**Table 2:** Selected properties of the tannery wastewater used for irrigation.

Parameters	Unit	Value
EC	(dS·m^−1^)	0.93
SAR	(mmol·L^−1^)^1/2^	3.42
RSC (Residual sodium carbonate)	(mmol_c_·L^−1^)	235
Cd	(mg·L^−1^)	0.02
Co	(mg·L^−1^)	0.04
Ni	(mg·L^−1^)	0.11
Cr	(mg·L^−1^)	2.86
Zn	(mg·L^−1^)	2.09
Bicarbonate	(mEq·L^−1^)	0.6
Oil and grease	(mg·L^−1^)	12
COD	(mg·L^−1^)	2916
TOC	(mg·L^−1^)	990
K	(mg·L^−1^)	43
Ca + Mg	(mEq·L^−1^)	3.3
